# Salivary oxytocin in autistic patients and in patients with intellectual disability

**DOI:** 10.3389/fpsyt.2022.969674

**Published:** 2022-11-24

**Authors:** Yulia A. Pichugina, Irina V. Maksimova, Marina A. Berezovskaya, Natalya A. Afanaseva, Aleksey B. Pichugin, Diana V. Dmitrenko, Elena E. Timechko, Alla B. Salmina, Olga L. Lopatina

**Affiliations:** ^1^Department of Psychiatry and Narcology, Prof. V.F. Voino-Yasenetsky Krasnoyarsk State Medical University, Krasnoyarsk, Russia; ^2^Social Neuroscience Laboratory, Prof. V.F. Voino-Yasenetsky Krasnoyarsk State Medical University, Krasnoyarsk, Russia; ^3^Department of Medical Genetics of Clinical Neurophysiology, Institute of Postgraduate Education, Prof. V.F. Voino-Yasenetsky Krasnoyarsk State Medical University, Krasnoyarsk, Russia; ^4^Medical Genetic Laboratory, Prof. V.F. Voino-Yasenetsky Krasnoyarsk State Medical University, Krasnoyarsk, Russia; ^5^Laboratory of Experimental Brain Cytology, Department of Brain Studies, Research Center of Neurology, Moscow, Russia; ^6^Research Institute of Molecular Medicine and Pathobiochemistry, Prof. V.F. Voino-Yasenetsky Krasnoyarsk State Medical University, Krasnoyarsk, Russia; ^7^Department of Biochemistry, Medical, Pharmaceutical and Toxicological Chemistry, Prof. V.F. Voino-Yasenetsky Krasnoyarsk State Medical University, Krasnoyarsk, Russia

**Keywords:** oxytocin, intellectual disability, behavior, childhood age, autism spectrum disorders

## Abstract

**Background:**

Assessing the role of oxytocin (OT) in the regulation of social interaction is a promising area that opens up new opportunities for studying the mechanisms of developing autism spectrum disorders (ASD).

**Aim:**

To assess the correlation between the salivary OT level and age-related and psychopathological symptoms of children with intellectual disability (ID) and ASD.

**Methods:**

We used the clinical and psychopathological method to assess the signs of ASD based on International Classification of Diseases (ICD-10), the severity of ASD was specified by the selected Russian type version “Childhood Autism Rating Scale” (CARS). Patients of both groups had an IQ score below 70 points.

**Results:**

The median and interquartile range of salivary OT levels in patients with ID and ASD were 23.897 [14.260–59.643] pg/mL, and in the group ID without ASD - Me = 50.896 [33.502–83.774] pg/mL (*p* = 0.001). The severity of ASD on the CARS scale Me = 51.5 [40.75–56.0] score in the group ID with ASD, and in the group ID without ASD—at the level of Me = 32 [27.0–38.0] points (*p* < 0.001). According to the results of correlation-regression analysis in the main group, a direct correlation was established between salivary OT level and a high degree of severity of ASD Rho = 0.435 (*p* = 0.005). There was no correlation between the salivary OT level and intellectual development in the group ID with ASD, Rho = 0.013 (*p* = 0.941) and we have found a relationship between oxytocin and intellectual development in the group ID without ASD, Rho = 0.297 (*p* = 0.005). There was no correlation between salivary OT and age, ASD and age.

**Conclusion:**

The results of this study indicate that patients in the group ID with ASD demonstrated a lower level of salivary OT concentration and a direct relationship between the maximum values of this indicator and the severity of autistic disorders, in contrast to patients in the group ID without ASD.

## Introduction

Oxytocin (OT) is a neuropeptide with two variants of hormonal effects: central and peripheral. OT has received much attention recently and has been called the “most studied neuropeptide” (1). OT is synthesized in the hypothalamus and released to the brain *via* distributed neural pathways and to the systemic circulation *via* the posterior pituitary (2). The peripheral effect of OT, associated with the regulation of labor and lactation is the most studied and actively used in medical practice.

The actions of oxytocin in the brain are attracted the attention of researchers with its influence on social behavior, parent-child relationships, stability of intra-group relationships, and communications (3–6). Plasma OT concentrations have been associated with gender, age, anxiety, attachment behavior, and cognitive abilities (7). There are studies that have shown that children who receive enough maternal care, physical, non-verbal and verbal contact, demonstrate higher levels of OT (8). The results of the study of low oxytocin levels support the impression that this neuropeptide actively affects the manifestations of mental disorders. There was a significant decrease in OT in women with postpartum depression (9, 10) with manifestations of alexithymia and anorexia nervosa (11, 12).

Nowadays, OT is a new biomarker and indicator of mental illness or the severity of the disease. Obtaining central OT levels is challenging, therefore researchers acquire peripheral OT levels through blood plasma or saliva, thereby offering an accessible way to assess on central OT circuitry. The meta-review of 2021 showed the significance of the dysfunction of the OT system in the mental illness (13). Another meta-analysis of 2021 has investigated whether there are differences in endogenous OT concentrations in individuals with ASD compared to matched neurotypical controls across all ages. It was showed OT levels are lower in children with ASD as compared to neurotypical controls, but this effect seems to disappear in adolescent and adult populations. OT levels of individuals with vs. without ASD seems to be only present in the studies with male participants. It was supposed that differences of OT levels were slightly more pronounced for blood samples as compared to saliva samples. Authors discussed that further research employing more homogeneous methods is necessary to explore the possible use of OT level measurement as a diagnostic marker of ASD (14). These results indicate the need to consider the age of patients when planning a research study to determine the level of endogenous oxytocin, giving preference to children of preschool and primary school age. Based on these data, a further study with more focus on pre-pubertal children on a cohort is therefore suggested. The same review drew attention to the important issue of comparing OT concentration levels in different environments and the significance of such differences for the diagnosis of mental disorders.

### Oxytocin level in central nervous system, saliva and plasma

The OT level is a very dynamic indicator with a rapidly changing concentration in the biological environments of the body. The oxytocin molecule has a very short half-life period [3–6 min on the periphery and 19 min in the central nervous system (CNS)], and the baseline level of OT in adults is quite low (<8 pg/ml) under initial conditions (15).

One of the studies, conducted using the method of liquid chromatography-mass spectrometry (LC-MS), showed that the maximum concentrations of this neuropeptide in urine, saliva and plasma were reached 15–30 min after intranasal administration with a sharp decrease over the next 2–3 h (16).

The information on the basic level of OT varies significantly in publications depending on gender, age and type of activity (cooking, playing different games individually or in groups etc.). In 31 students, the concentration of the hormone in saliva was recorded before the study 45.96 (33.27) – 77.93 (74.46) pg/ml (17); there was a difference in the level for different variants of activity (individual and group) from 60 ± 8 to 234 ± 35 pg/ml in women and from 63 ± 15 to 207 ± 24 pg/ml in men (18).

For children with typical development, the OT level is 90–100 pg/ml, which differs from the OT level in children with the consequences of abuse (19). Some authors show normalized values of the OT level, which makes it difficult to find information about absolute indicators of the OT level in saliva (20).

Recent investigations show that research opportunities in reliably assessing the effect of OT on humans are significantly limited by the inaccessibility of CNS structures. In this regard, it becomes relevant to use alternative samples as a source for assessing the activity of the hormone in the human brain, which makes it possible to eliminate the risks associated with the intake of cerebrospinal fluid (CSF). Urine and saliva are the preferred mediums for OT analysis because of their non-invasive collection methods (21). Although there are many publications indicating that it is necessary to clarify the correspondence of neuropeptide levels in various biological fluids (22) due to conflicting views on the relationship between central and peripheral OT activity. There are studies about the degree of correlation of OT in plasma, saliva and CSF (23–25). A study of the OT concentration in biological samples of patients with neurological and neurosurgical pathology showed a significant correlation between saliva and CSF, thereby confirming that saliva OT can help assess the level of OT in the CNS. On the contrary, the OT level in plasma did not correspond to its level in the CSF (13, 22).

### Oxytocin and autism spectrum disorders

In accordance with the present results, previous studies have demonstrated OT role in the mechanisms of regulation of social behavior, adaptability and emotional response in patients with ASD. This finding broadly supports the work of other studies in this area and indicate that OT can be considered a good diagnostic marker in patients with severe autism. Changes in plasma levels of neuropeptides, including OT and mRNA, correlated with the severity of disorders, have been reported in children with ASD (26).

It was shown that OT levels (44.72 ± 36.1 pg/ml) in children with ASD were significantly lower (*p* < 0.05) than in age-matched (102.1 ± 34.31 pg/ml) and sex-matched (53.05 ± 38.38 pg/ml) children of the control group. In the same study, a noticeable correlation was noted between the severity of developmental disorders and the concentration of the hormone in saliva: for mild degree—59.22 ± 27.32 pg/ml, for moderate degree—47 ± 25.47 pg/ml, for severe degree—27.92 ± 10.23 pg/ml (26).

The level of endogenous OT is associated not only with the severity of ASD, but also with changes in the functioning of brain regions. The identified associations between the oxytocinergic system and amygdala–hippocampal pathways are anticipated to be of relevance for understanding the role of OT in modulating appropriate neural and physiological responses to stress and restoring homeostasis. It was revealed that in adult men with ASD, higher levels of salivary OT are associated with higher expressions of secure attachment but lower degrees of interregional functional coupling between the amygdala and hippocampal regions (27).

Measurement of the OT level in patients with ASD was performed to assess its role in the systematic regulation of socialization, monitor the results of the use of intranasal OT spray, study the impact on cognitive abilities and emotional attachment (28–30). The significant correlation in the whole-group analysis was presumed to reflect the fact that the ASD subgroup showed abnormal peripheral OT levels. Based on the spectrum concept described above, the salivary OT level reflects the ASD characteristics in the whole-group analysis. Conversely, the significant correlations between salivary OT level and AQ (The Autism Spectrum Quotient) were not observed in the within-group analysis (31).

The plasma levels of these neuropeptides reflected different aspects of autistic behaviors, with OT correlated with verbal communication. Thus, the dependence of the OT concentration and the manifestations of the verbal communication violations were noted in a group of 84 patients with ASD compared with a group of 85 participants without ASD. Correlation analysis showed that children with ASD with higher plasma OT concentrations tended to have less impairment in verbal communication (Rho = –0.22, *P* = 0.076) and had lower plasma OT levels than neurotypical children of the corresponding gender (*P* = 0.028) (32).

Working with young patients does not always allow taking blood for research; therefore saliva sampling is a non-invasive method for obtaining a biological sample. The concentration of neuropeptides in saliva is no less informative and reliable than level of neuropeptides in blood plasma. It was found that salivary OT increased in children with ASD during interaction with parents and decreased 15 min after the end of contact (20).

Importantly, pretreatment blood OT concentrations also predicted treatment response, such that individuals with the lowest pretreatment OT concentrations will be showed the greatest social improvement. It was found that the response to therapy was directly related to the initial OT level. So OT treatment enhances social abilities in children with ASD and that individuals with pretreatment OT signaling deficits may stand to benefit the most from OT treatment. These results indicate that peripheral OT levels may reflect the degree of social deficit, and that it is important to understand how CNS and plasma, plasma and salivary OT levels are related to the severity of ASD. This rather contradictory result may by due to bias and cannot be extrapolated to all patients. Results should be interpreted with caution. So in one study of the use of intranasal OT in children with ASD, an association was shown between social improvement and an increase in the level of the hormone in plasma in children who received placebo (33). Another study reported similar OT levels in healthy people and autistic people [ASD 36.2 ± 13.2 pg/mL, TD 43.6 ± 17.0 pg/mL, t(38) = 1.45, *p* = 0.154, Cohen’s *d* = 49] in studying the relationship between attention to social information and peripheral levels of OT (31).

All this demonstrates the need to refine the options for implementing the effects of OT and to search for factors that create conditions for obtaining more reliable results.

### Oxytocin and autism spectrum disorders with intellectual disability

There is practically no information about the level of OT in people with intellectual disability (ID). At the same time, a large number of people with ASD have ID (34). There is evidence that the functions of OT differ in men and women with severe mental retardation and that OT partially affects autism and is associated with some repetitive actions and non-verbal communication in patients with ASD with severe mental retardation (35).

Some research results show that oxytocin can improve social interaction in a group of patients suffering from ASD with concomitant ID (36). It remains unclear whether the presence of mental retardation affects the level of OT in ASD. This is important to understand for the development and application of treatments for ASD based on changes in OT levels.

### Hypothesis

We hypothesized based on existing evidence that patients with ID with ASD and patients with ID without ASD would have different levels of OT in saliva. This hypothesis is based on the fact that a reduced level of OT is characteristic of ASD (26), that OT in saliva reflects the level of OT in the CNS better than OT in plasma (22).

The aim of the study: to assess the correlation of salivary OT levels with age and psychopathological characteristics of children suffering from ID and ASD.

## Materials and methods

### Patients

The study included 121 children hospitalized for the first time in the Pediatric Department of the Krasnoyarsk Regional Psychoneurological Early Treatment Center, Krasnoyarsk, Russia, which was an approved platform for participation in the research of Krasnoyarsk State Medical University.

The children underwent inpatient treatment between 2018 and 2021 for aggressive, self-harming behavior, and emotional problems. The respondents were selected by the consent of legal representatives to participate in the study, the age of 4–13 years, the presence symptoms of ID, of ASD, the ability to complete tasks (Human figure drawing test and spitting saliva into a test tube). Patients over the age of 13 were excluded due to the high probability of the influence of hormonal characteristics. We included children with disabilities (the patients are given a disability group) and orphans in the study in compliance with all the rules for obtaining consent and monitoring compliance with their rights (Tables 1, 2).

**TABLE 1:** Descriptive statistics of quantitative variables.

Indicators	Me		Q_1_ – Q*3*		Min		Max		*ETH*
	ID with ASD (*n* = 34)	ID without ASD (*n* = 87)	ID with ASD (*n* = 34)	ID without ASD (*n* = 87)	ID with ASD (*n* = 34)	ID without ASD (*n* = 87)	ID with ASD (*n* = 34)	ID without ASD (*n* = 87)	
Age	7.0	9.0	5.75–10.0	6.0–11.0	4	3	12	13	*p* = 0.098
IQ^a^	61.0	63.0	45.8–70.0	50.0–68.0	37	37	70	70	*p* = 0.589
CARS^b^	**51.5**	**32.0**	**40.8–56.0**	**27.0–38.0**	33	13	59	51	***p* < 0.001**

ID with ASD—a group of patients with intellectual disability and autism spectrum disorders; ID without ASD—a group of patients with intellectual disability without autism spectrum disorders.

IQ^a^ indicators of the intellectual level, determined using the test “Human figure drawing test”.

CARS^b^ Childhood Autism Rating Scale.

Me [Q1—Q3]-Median [25–75% percentile], the first quartile, Q_1_, is equal to the 25th percentile, the third quartile, Q_3_, is equal to the 75th percentile.

Min, max—minimum, maximum values of indicators. The bold values indicate values with *p* < 0.001.

**TABLE 2:** Descriptive statistics of categorical variables.

Indicators	Abs.		%			
	ID with ASD (*n* = 34)	ID without ASD (*n* = 87)	ID with ASD (*n* = 34)	ID without ASD (*n* = 87)	χ^2^	*P*
Sex male/female	27/7	66/21	79.4/20.6%	75.9/23.1%	0.173	*p* = 0.677
Orphancy	**1**	**26**	**2.9%**	**29.9%**	**10.238**	***p* = 0.001**
The patients are given a disability group	**16**	**16**	**47.16%**	**18.39%**	**10.329**	***p* = 0.001**

ID with ASD—a group of patients with intellectual disability and autism spectrum disorders; ID without ASD—a group of patients with intellectual disability without autism spectrum disorders. The bold values indicate values with *p* = 0.001.

In the ID with ASD (Pervasive developmental disorders F84) group, there were 8 children with Childhood autism (F84.0), 12 children with Atypical autism (F84.1), 9 children with Mental retardation with autistic features (F84.1), 1 with Rett syndrome (F84.2), 4 children with Other childhood disintegrative disorder (F84.3). In the group ID without ASD, there were 68 children with Mild mental retardation (F70.0–F70.1), 19 children with Moderate mental retardation (F71.0–F71.1).

Most of the patients from both groups received antipsychotics, antidepressants, nootropic drugs, according to clinical guidelines and treatment protocols. None of the study participants received psychostimulants.

Almost all children received help from a psychiatrist at their place of residence in specialized outpatient clinics for children and adolescents before hospitalization. There was no information about receiving specialized psychiatric care before admission to the hospital in 1 child from the group ID with ASD and in 9 children from the group ID without ASD. The organization of treatment both in the polyclinic at the place of residence and in the hospital was necessarily accompanied by psychological work and psychoeducation of the patients themselves, their parents, teachers to improve socialization, facilitate adaptation in the children’s collective, and reduce suffering.

### Informed consent

The parents of the patients signed a written consent prior to inclusion on the basis of complete information in accordance with the requirements of the “Law of the Russian Federation on July 2, 1992 N 3185-1 on psychiatric care and guarantees of the rights of citizens in its provision.”

In the absence of parents, consent was obtained from legal representatives:

– close relatives who have the official status of guardians appointed by the court of the Russian Federation to represent the interests of a child left without parental care;
– an official from the institution of residence of a child authorized by the state to monitor the observance of the rights and interests of an orphan child who is on full state support and has neither parents nor guardian relatives.

Children under the age of 13 gave their verbal consent.

Separately, written consent was obtained for medical intervention to determine the level of OT and consent to the processing of personal data. The study was conducted in compliance with the modern biomedical ethics and ethical standards developed in accordance with the World Medical Association Declaration of Helsinki (Protocol No. 86/2018 of the local ethical committee of Krasnoyarsk State Medical University of 08.11.2018).

### Methods

The analysis of the clinical picture of disorders based on diagnostic criteria (ICD-10) was performed in all children. The signs of mental disorder were studied by two psychiatrists. The first was an employee of the hospital, the second—an employee of the Medical University. Further, the results of the clinical examination were supplemented with materials of experimental psychological examination and psychometric methods. This stage was implemented by the staff of the psychological service of the hospital.

### Assessment of the severity of autistic disorders

The method “Childhood Autism Rating Scale (CARS)” (37, 38) made it possible to assess and compare the degree of social and communicative deficit in patients with soma of various pathologies. In the works devoted to the study of the validity of this test, its reliability was demonstrated. The Cronbach’s alpha coefficient for CARS was 0.772, and the degree of correlation with the Autism Behavior Checklist (ABS)—0.732 (39). Information that CARS may not provide a cross-cultural valid assessment of ASD (40), pointed out the need to use a proven adapted version of the test (41). The adapted Russian version showed fairly high reliability values of *r* = 0.9 (*p* < 0.001) and correlations with the scale of the CARS *r* = 0.8 (*p* < 0.001) (41).

In addition, the CARS technique could not be used independently for the diagnosis of ASD. Many of the signs listed in it could be observed in other diseases. This type of examination required a mandatory assessment of the compliance of the condition with the criteria of ICD-10 by a psychiatrist.

### Determining the degree of intellectual disability

We used the “Human figure drawing test” to assess the intellectual level, which made it possible to correlate the degree of development of cognitive functions with a 100-point IQ-test system. To date, this diagnostic procedure is quite unified, widely used for intellectual assessment, is readily used in medical institutions, schools, medical schools, and social rehabilitation centers (42, 43). There is an option to compare the results of autism research conducted in various fields of health and education. When studying the validity of the “Human figure drawing test” in children with mental disorders, a general correlation of the Full Scale Intelligence Quotient (FSIQ) from the standard intellectual level test and standard Goodenough-Harris scores corresponding to 0.813 (*p* < 0.01) was established. For patients with an intelligence quotient (IQ) of less than 70 points, the validity of the test was 69.2% (44). The “Human figure drawing test” offered an effective way to the tasks of our study. In some patients included in the survey, it was impossible to determine the intellectual level using a full-scale version of the IQ-test due to the autistic characteristics of children or their behavioral disorders. The drawing test was convenient, performed within a few minutes, well understood, did not provoke protest emotional and behavioral reactions in the survey participants (45).

### Saliva sampling and oxytocin measurement

Saliva sampling was performed to assess the hormonal status and determine the level of OT. Saliva sampling was carried out on the 7–10th day of hospitalization, in the morning from 8 to 9 o’clock, before meals. Saliva samples (0.3–0.8 ml) collected in paper cups were immediately transferred to plastic microtubes (1.5 ml). They were frozen and stored at –20°C to be centrifuged twice at 4°C at 1,500 × *g* for 15 min 3 days later. The samples were kept at –20°C until assayed.

Determination of salivary OT was performed using a 96-plate commercial OT-ELISA kit (Clode-Clone Corp., China), as described previously (46). Recent studies have shown that OT values are reliable when measure in saliva by enzyme immunoassay (46, 47). Measurements were performed in duplicate. Samples (100 μl) were treated according to the manufacturer’s instructions. The optical density of the samples and standards was measured at wavelengths of 450 nm by a microplate reader. Sample concentrations were calculated according to the relevant standard curve (48).

Several studies have shown that OT in saliva, determined by immunoassay, is reliable, stable over time results, and correlates with the behavioral processes (49, 50).

### Statistical analysis

We carried out the mathematical analysis using the SPSS medical statistics software package for Windows version 24. The significance of the results was established at *p* < 0.05 and *p* < 0.01. The normality of the distribution was checked using the Shapiro-Wilk test.

Analyzing the deviation from the normal distribution of features, the descriptive part of statistical processing was carried out by non-parametric methods. Medians and interquartile range (Me [25%; 75%]) were calculated for indicators of quantitative variables of age, the severity of autism, the severity of ID, the salivary OT level. Categorical variables of indicators of gender, disability, and orphanhood were evaluated using the Pearson Criterion (χ^2^).

Spearman’s correlation coefficients (Rho) were determined in each group to assess the correlation of ASD symptoms with age, OT and the ID degree.

We used multivariate regression analysis to predict the degree of influence of independent variables (constant—study group, gender, age, intelligence, CARS, orphanhood, disability) on the dependent variable OT (salivary oxytocin level). Regression analysis was carried out both for the combined database and separately for the group ID with ASD and for the group ID without ASD.

The sample size of ID patients with ASD *n* = 34 for cross-group comparisons was determined with an estimated effect size of 0.35; alpha = 0.05; degree = 0.84 (G*Power3.1.9.7).

## Results

**FIGURE 1:**
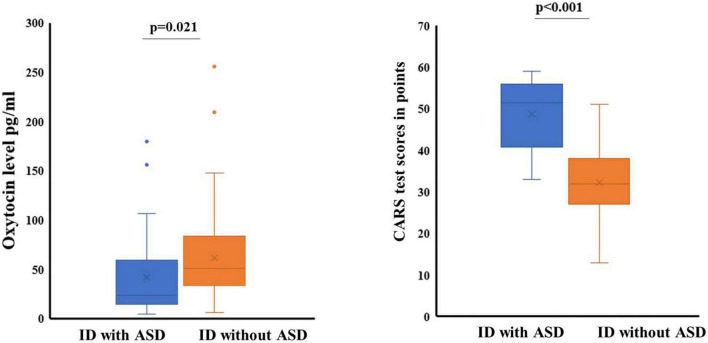
The concentration of OT in saliva, CARS test score and the level of severity of ASD. ID with ASD—the group of patients with intellectual disability and autism spectrum disorders; ID without ASD—the group of patients with intellectual disability without autism spectrum disoders.

We did not find any significant differences between the groups in the ratio of sex (*p* = 0.678), age (*p* = 0.098) or IQ level assessed by the “Human Figure drawing test” (*p* = 0.589). Differences were found in the levels of OT concentration in saliva and indicators of the severity of autism between the groups (Figure 1).

Median indicators and interquartile range (ME [Q_1_-Q_3_]) of OT level in saliva of patients in the group ID with ASD were 23.897 [14.260–59.643] pg/ml, and in the group ID without ASD were Me = 50.896 [33.502–83.774] pg/ml. The difference between the indicators has reached the required level of significance (*p* = 0.001). Our results of assessing the severity of ASD on the CARS scale showed Me = 51.5 [40.75–56.0] score in the group ID with ASD. For patients from the group ID without ASD, the severity of ASD was at the level of Me = 32 [27.0–38.0] points, difference (*p* < 0.001).

Our results revealed a significant difference between the groups in terms of the number of orphans and the disabled. 29.9% of children from the group ID without ASD showed signs of social distress due to orphanhood. In the group ID with ASD, only 1 child (2.9%) was an orphan (*p* = 0.001). Children from group ID with ASD had given a disability group in 47.1% compare with 18.4% of patients with ID without ASD (*p* = 0.001).

**FIGURE 2:**
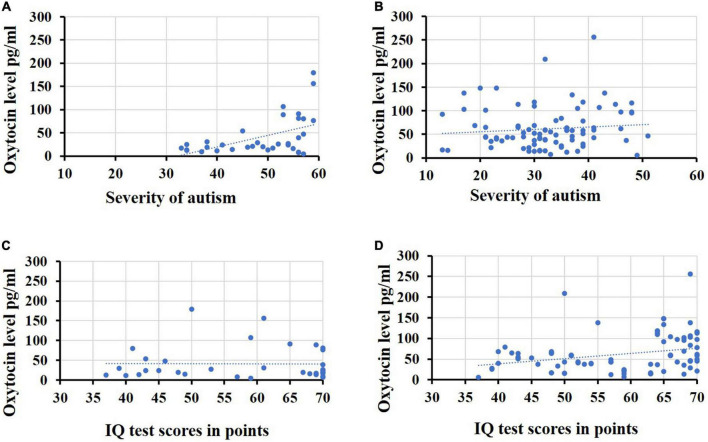
Indicators of the oxytocin level. **(A)** The oxytocin level in saliva and severity of autism in the group ID with ASD, **(B)** the oxytocin level in saliva and severity of autism in the group ID without ASD, **(C)** the oxytocin level in saliva and the level of intellectual development patients in the group ID with ASD, **(D)** the oxytocin level in saliva and the level of intellectual development patients in the group ID without ASD.

The results of the correlation analysis performed by us showed that patients from the group ID with ASD maintained a direct relationship between indicators of severe severity of ASD and high levels of OT concentration in saliva (Figures 2A,B). Spearman’s correlation coefficient in this group was Rho = 0.435 (*p* = 0.005). In the second group of ID without ASD, this relationship was not significance Rho = 0.104 (*p* = 0.338). At the same time, we did not find a correlation between the level of OT concentration in saliva and the level of intellectual development for the ID group with ASD, Rho = 0.013 (*p* = 0.941) and we have found of a relationship in OT indicators with intellectual development for the ID group without ASD, Rho = 0.297 (*p* = 0.005) (Figures 2C,D).

**FIGURE 3:**
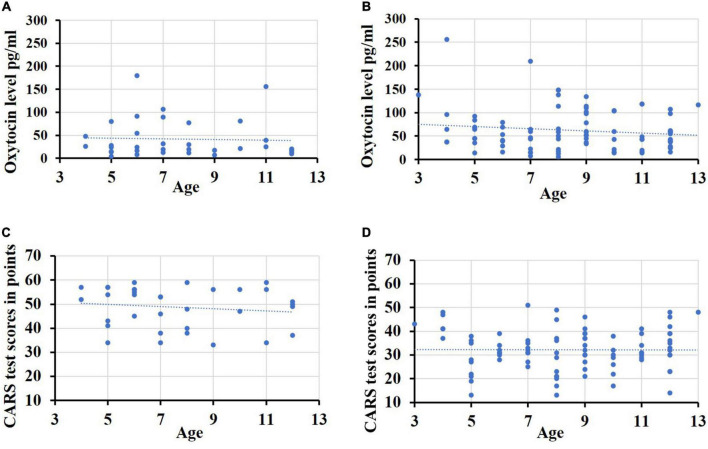
Indicators of severity of autistic disorders, oxytocin levels and age. **(A)** The oxytocin-levels and age in the group ID with ASD; **(B)** the oxytocin levels and age in the group ID without ASD; **(C)** the severity of autistic disorders and age in the group ID with ASD; **(D)** the severity of autistic disorders and age in the group ID without ASD.

There was no significant correlation between age and the salivary OT (Figures 3A,B) in both groups. The correlation coefficient in the ID group with ASD was Rho = –0.087 (*p* = 0.623), and in patients and the ID group without ASD - Rho = –0.076 (*p* = 0.481). The results of assessing the relationship between the severity of autistic signs and age did not reach the required level of significance (Figures 3C,D). The correlation coefficient in the ID group with ASD was Rho = –0.182 (*p* = 0.302), and in patients and the ID group without ASD – Rho = 0.013 (*p* = 0.903).

To clarify the degree of influence of factors, a multiple regression analysis was carried out. We assessed the degree of change in the dependent variable (indicators of OT concentration in saliva), under the influence of several factor signs (gender, age, severity of autism, severity of intellectual disability, the state of orphanhood and the presence of disability).

The regression model we constructed showed a moderate Chaddock scale correlation (weak—from 0.1 to 0.3; moderate — from 0.3 to 0.5; noticeable—from 0.5 to 0.7) for the combined groups between the concentration of oxytocin in saliva and other factors (*R* = 0.376). This model will be correct for 14% of cases.

The calculation of a separate model for each group showed the differences between them. For the group ID with ASD, a significant Chaddock scale correlation was established between the salivary OT concentration and other factors (*R* = 0.586), and for patients from the group ID without ASD, a moderate correlation was established (*R* = 0.33). The probability of error in predicting results in this model also differed between groups. For the group ID with ASD, the constructed model is correct in 34% of cases (*R*^2^ = 0.343), and in the group ID without ASD, our model of the influence of several factors on the concentration of OT in saliva is correct only in 10% of cases (*R*^2^ = 0.108).

**FIGURE 4:**
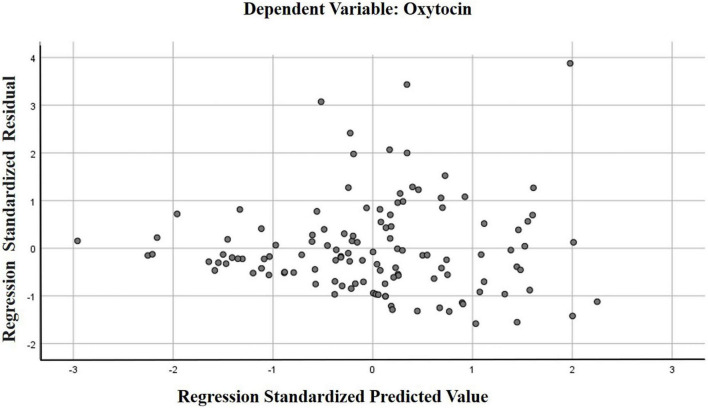
Scatter plot of standardized predicted values against standardized residuals for the regression model with combined groups.

**FIGURE 5:**
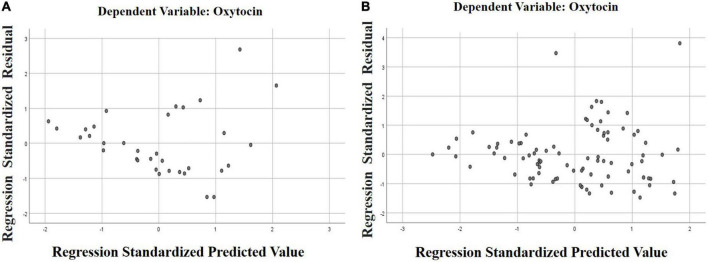
Scatter plot of standardized predictive values versus standardized residuals for each group’s regression model. **(A)** ID with ASD (the group of patients with intellectual disability and autism spectrum disorders); **(B)** ID without ASD (the group of patients with intellectual disability without autism spectrum disorders).

According to the values of the coefficient B, we found that of all the indicators used in the model, a significant effect on the dependent variable (the indicator of the saliva OT concentration). This has been confirmed for the severity of ASD on the CARS scale, B = 0.987 (*p* = 0.039) in the model with combined groups. In the model with the group ID with ASD, a reliable level of indicators of the influence of the severity of ASD on the concentration of OT in saliva remained, as in the model with the combined groups, B = 2.959 (*p* = 0.002). In the model with the group ID without ASD, reliable indicators of the effect on the concentration of OT in saliva were found for IQ indicators, B = 1.167 (*p* = 0.018). The results of multiple regression analysis are presented in Table 3 and Figures 4, 5.

**TABLE 3:** Summary of hierarchical regression analysis for patients in both groups.

	Combined indicators		ID with ASD (*n* = 34)		ID without ASD (*n* = 87)	
	Unstandardized coefficients B	*p*	Unstandardized coefficients B	*p*	Unstandardized coefficients B	*p*
Constant	–78.723	0.051	–70.937	0.161	–1.280	0.971
Group	42.931	0.001*	–	–	–	–
Gender	–5.617	0.562	–24.527	0.182	–2.307	0.840
Age	–1.757	0.287	4.433	0.233	–1.956	0.309
CARS	0.987	0.039**	2.959	0.002**	0.427	0.452
IQ	0.711	0.073	–1.065	0.180	1.167	0.018**
Orphanhood	–11.891	0.259	–41.247	0.330	–8.098	0.467
Disability	5.898	0.530	5.510	0.695	3.470	0.778
*R*—determination coefficient	0.376		0.586		0.330	
*R*^2^—the value of the coefficient of determination	0.141		0.343		0.108	
*F*	2.661		2.35		1.627	

*p = 0.001, **p < 0.05. The bold values indicate values with significant difference.

## Discussion

The data we have obtained require discussion and further study. The results of our research, which showed a lower saliva OT level in children grope ID with ASD, correspond to the results of other studies studying the nature of the dysfunction of the OT system in this pathology (51).

At the same time, the direct correlation that we established between the indicators of severe autistic disorders and high levels of saliva OT concentration in the group ID with ASD does not correspond to the materials of scientific publications on the reduced concentration of this hormone in autistic pathology (52). Such a result may be associated with different levels of adaptive abilities of patients, or it may be due to the peculiarities of the response to stress and the more significant than expected role of OT in the regulation of response to emotional and intellectual stress.

The stressful situation of the examination can provoke the release of OT due to the fear and anticipation of subsequent difficulties (53), which contributes to the reduction of harm from stress due to cardioprotective mechanisms of OT that effect the magnitude and hemodynamic parameters of cardiovascular stress responses (54).

It is likely that OT can increase physical and cognitive resilience, preventing the consequences and improving the clinical outcomes of stress-related traumatic disorders after acute or prolonged traumatic experiences (55). At the same time, there is information that OT improves impaired social adaptation by normalizing hyper- or hypo-brain activity (56) and demonstrates an adaptive effect in both autistic and neurotypical study participants (52).

It is necessary to plan the application of methods for assessing adaptability and stress resistance in further studies.

The low rates of orphanhood and elevated OT levels that we found in the group of ID with ASD with more severe variants of developmental disorders that required social support in the form of the given a disability group, deserve attention and additional study. The reliable difference between the groups in these parameters, established by using descriptive statistics methods, was significantly limited during further processing. Multiple regression analysis did not reveal the correlation and mutual influence of these indicators. However, the presence of significant differences between the groups and the assumption that it is the increase in the content of this hormone in biological environments that affects the stability of emotional ties, reducing the risk of parents abandoning a sick child, will remain relevant to research in this area.

Based on the identified cases of high OT levels in the group ID with ASD in severe variants of ASD, we can safely assume that the level of OT indicates not so much the severity of disorders as the preservation of adaptive mechanisms. This assumption is supported by the materials of studies on the influence of OT on parent-child relationships, for the implementation of protective mechanisms in painful procedures (57), on attention to social signals (58).

While OT significantly affects the mechanisms of social adaptation (56), it seems that the effects of OT strongly depend on the context and experience gained, especially at an early age (59).

Patients with higher indicators of intellectual development in the group ID without ASD are easier than other participants to record and use positive experiences, realizing it through mediated mechanisms and reactions accompanied by an increase in the level of OT in saliva (60).

In addition, other hypotheses and explanations are acceptable.

Taking this into account, in further studies it is necessary to plan the use of methods for assessing adaptability and stress resistance. The use of medication by our participants is a limitation of the study. Published articles contain indications of such limitations (33). Today, it is not known how drugs interact with OT and change its concentration. The necessary conditions were ensured by the fact that during the study the drug intake did not change, remained stable and did not differ between groups.

There are practically no patients with ASD and high intellectual level in the hospital. As a rule, such patients receive the necessary assistance and support at their place of residence and study. The hospital approved for the study is referred by a psychiatrist from the place of residence. These are patients with the most severe variants of pathology. We formed the study groups from such patients.

The study of neurotypical children and children with ASD and high intellectual level requires the organization of work in other conditions, for instance, at school. This is beyond our capabilities and leads to limitations in research.

The relatively small sample size is also a potential limitation. We conducted a study among patients and their relatives who have a higher motivation in organizing treatment due to fairly severe disorders. We cannot directly transfer the results obtained to all patients with ASD or ID.

The research outcomes and the limitations described indicate the relevance of organizing studies with the required number of patients. Despite the complexity and rather high cost of such work, which make it difficult to create a large sample, it is necessary to conduct research on more powerful cohorts to deepen our understanding of the mechanisms of oxytocin’s influence on the pathogenesis of autism spectrum disorders.

## Conclusion

As a result of the study, we found that children with ASD, who were admitted to the hospital for the first time, revealed a lower salivary OT level compared to the group of patients with ID. Children with autistic features showed a more noticeable tendency to increase OT levels in severe ASD than children with ID. For patients of the group ID without ASD, a higher level of OT concentration in saliva and a direct relationship of the maximum values of this indicator with a milder degree of ID were established. Although these results are correlational and preliminary, it raises several intriguing ideas that deserve further study.

## Data availability statement

The original contributions presented in this study are included in the article/supplementary material, further inquiries can be directed to the corresponding author.

## Ethics statement

The studies involving human participants were reviewed and approved by the Local Ethical Committee of Krasnoyarsk State Medical University. Written informed consent to participate in this study was provided by the participants’ legal guardian/next of kin.

## Author contributions

YP: literature collection and analysis, concept and design development, data interpretation and analysis, verification of critical content, and preparation of the manuscript. IM and ET: data interpretation and analysis and preparation of the manuscript. MB and DD: data interpretation and manuscript substantiation. NA: collecting material. AP: collecting material, data processing, and analysis. AS: verification of critical content. OL: concept and design development, literature collection and analysis, data processing, and verification of critical content. All authors contributed to the article and approved the submitted version.
